# recoup: flexible and versatile signal visualization from next generation sequencing

**DOI:** 10.1186/s12859-020-03902-x

**Published:** 2021-01-06

**Authors:** Panagiotis Moulos

**Affiliations:** Institute for Fundamental Biomedical Research, Biomedical Sciences Research Center ‘Alexander Fleming’, Fleming 34, 16672 Vari, Greece

**Keywords:** Next generation sequencing, Signal visualization, Transcription factors, ChIP-Seq, ATAC-Seq, RNA-Seq, Genomic profiles

## Abstract

**Background:**

The relentless continuing emergence of new genomic sequencing protocols and the resulting generation of ever larger datasets continue to challenge the meaningful summarization and visualization of the underlying signal generated to answer important qualitative and quantitative biological questions. As a result, the need for novel software able to reliably produce quick, comprehensive, and easily repeatable genomic signal visualizations in a user-friendly manner is rapidly re-emerging.

**Results:**

recoup is a Bioconductor package for quick, flexible, versatile, and accurate visualization of genomic coverage profiles generated from Next Generation Sequencing data. Coupled with a database of precalculated genomic regions for multiple organisms, recoup offers processing mechanisms for quick, efficient, and multi-level data interrogation with minimal effort, while at the same time creating publication-quality visualizations. Special focus is given on plot reusability, reproducibility, and real-time exploration and formatting options, operations rarely supported in similar visualization tools in a profound way. recoup was assessed using several qualitative user metrics and found to balance the tradeoff between important package features, including speed, visualization quality, overall friendliness, and the reusability of the results with minimal additional calculations.

**Conclusion:**

While some existing solutions for the comprehensive visualization of NGS data signal offer satisfying results, they are often compromised regarding issues such as effortless tracking of processing and preparation steps under a common computational environment, visualization quality and user friendliness. recoup is a unique package presenting a balanced tradeoff for a combination of assessment criteria while remaining fast and friendly.

## Background

The constant development of Next Generation Sequencing (NGS) techniques continues to expand the seemingly endless portfolio of applications in research, the clinic, and the industry. As a result, its current dominance in modern high-throughput molecular biology continues to challenge the ecosystem of current NGS data analytics solutions, creating the need for new tools capable of offering comprehensive, intuitive, and flexible visualizations under several experimental settings and factors. In addition, the ever-expanding library of emerging protocols at the bulk and single-cell level, including for applications such as protein-DNA interactions [1] and gene expression monitoring [2], produce larger datasets both in terms of size and scale. As a result, new needs arise for efficient data management, organization and process tracking, summarization, fast quality control, and simple and reproducible visualizations with minimized manual interaction.

An established analytical tool in NGS analysis protocols and usually the first contact point of the investigator interrogating the data, which often comprises the ground truth and sanity check for any subsequent analyses, is the visualization of the signal created by short reads after the alignment or assembly to a reference genome. Genome Browsers [3] serve this purpose very efficiently when single genomic areas are considered. However, they fail to visualize average signal profiles over multiple genomic locations and to depict such information in a comprehensive way. Such regions may include but are not limited to gene and DNA methylation regions, transcription factor binding sites and accessible chromatin loci. Furthermore, they cannot visualize average signal profiles of factored data, for example H4K20me3 methylation signals in a set of genes assigned to different expression levels; moreover, they cannot visualize the totality of signals of interest mapped on all the interrogated loci in a compact way, something achieved using a heatmap.

To create such visualizations, multiple manual steps are usually applied, where several toolkits and custom, often non-reusable scripts are deployed to import short reads, overlap them with genomic regions of interest, summarize them by binning and averaging read abundances to control the resolution of signals and finally render the graphics. While several solutions have been proposed to automate the aforementioned procedure to some extent [4–7], some suffer from poor visualization issues and the dependence on several manual data preparation steps, while others are complex to setup and use and therefore hinder reproducibility. Furthermore, even though some are accompanied by a Graphical User Interface (GUI), their usage is not intuitive for the inexperienced user and may additionally suffer from poor maintenance.

recoup fills these gaps by using standard Bioconductor facilities to process short reads and modern graphics systems to create comprehensive genomic signal profiles in a highly-automated fashion. In addition, it offers a plethora of customization options and visualization at various levels as well as facilities to manipulate and reuse genomic profiles involving lengthy calculations.

## Implementation

### Package overview

**Fig. 1:**
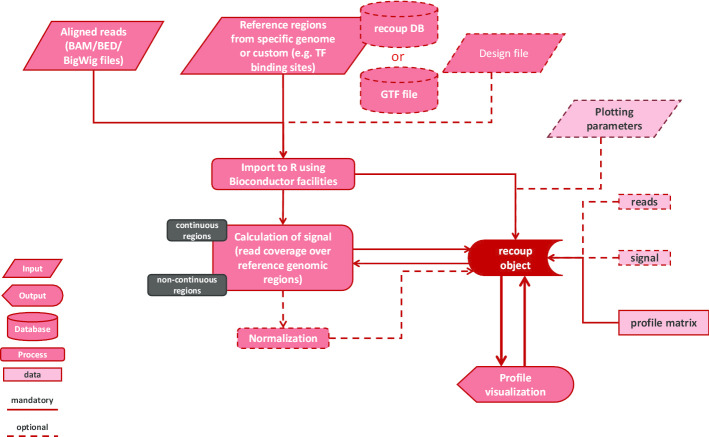
The recoup workflow: from raw aligned reads to reusable coverage profiles of high quality

recoup is a Bioconductor package which makes efficient use of parallelization in most signal calculation steps and Bioconductor functions and data structures to stream and manipulate short reads in various formats derived from genome-wide sequencing protocols. The genomic loci which are interrogated for signal levels can be provided either by the user as a BED-like file or from a database of pre-calculated genomic regions for a variety of use cases and several supported organisms. Signal profiles can be calculated in two modes: i) the coverage mode, where signal abundance is calculated based on read pileups either at single-base levels for maximum resolution, or summarized over bins of user-specified size to further speed-up calculations, ii) the Reads Per Million (RPM) mode, where reads are counted over dynamically sized bins spanning the interrogated genomic loci, with RPM for each region being reported for profile generation. Case (ii) is even faster at the cost of signal plot resolution and is ideal for high-level qualitative signal representations. Moreover, signal calculation operations including binning, normalization, and resolution control can be performed with a variety of supported methods. Upon signal computation, the results are stored in an object specifically designed to maximize reusability and minimize the required calculation steps in case of configuration changes or zooming-in, slicing, and sub-setting, operations performed through simple function calls. Average signal profile plots are rendered using the ggplot2 and ComplexHeatmap [8] graphics grammar systems. Plot faceting can be performed automatically per sample or given a set of biological conditions through a simple design file; k-means clustering of the profiles is also supported. Finally, more experienced users can adjust a wealth of additional options to maximize versatility and the plot outcomes can be further configured using ggplot2 facilities. The recoup workflow is depicted in Fig. 1. Additional descriptions of several functionalities can be found in Additional File 1.

### Visualization types

recoup creates three types of plots, all of which are based on a matrix of signals calculated from counting read pileups (signal per base or signal per bin of bases) or reads per million for each genomic region. All plot types can be calculated over two types of genomic regions (continuous and non-continuous). The following sections describe the plot and region types.

#### Average signal coverage plots

These plots consist of one or multiple curves, which are created by averaging (mean or median) the columns of one or more signal matrices and depict the average signal pattern over the regions of interest. A measure of dispersion (standard deviation or median absolute deviation) is also calculated and optionally displayed. The plots can be faceted according to several factors, including the provision of a design file, k-means clustering, sample-wise or a combination of these.

#### Signal heatmap plots

These plots depict the signal over the regions of interest *as is* and without any averaging over the signal matrices. recoup supports many further display options for the heatmaps, including faceting by a design file or k-means clustering and hierarchical clustering supported by the ComplexHeatmap [8] package. In addition, many options are supported for ordering the heatmap by signal, average signals over all samples or the signal from a specific sample. More details are provided in the package documentation.

#### Signal correlation plots

These plots comprise one or usually more curves which visualize potential correlations of signals between samples over the regions of interest. This is achieved by calculating average region-wise coverage values for each reference (row- or region-wise) in the signal profile matrices instead of the average coverage on each base or bin of the reference regions (column- or base/bin-wise). Dispersion of signals may also be displayed. This signal representation is particularly useful for checking whether total genome profiles for some biological factor/condition correlate with each other.

#### Continuous regions

In these genomic regions the signal is considered continuous in terms of coverage. Such regions may be of interest in protocols such as ChIP-Seq, ATAC-Seq, and Whole Genome Sequencing. In this case, the signal is plotted in a continuous manner, without stitching, as regions are not separated.

#### Non-continuous regions

In these genomic regions the signal is considered non-continuous in terms of coverage. Such regions may be of interest in RNA NGS applications or DNA-sequencing applications, such as Whole Exome Sequencing and targeted Gene Panel Sequencing. In this case, the signal is stitched prior to plotting, as covered regions are expected to be distant from each other (e.g. exons separated by intronic regions).

#### Additional options

Finally, many plot customization options are supported, providing both the novice and experienced recoup user with a variety of approaches to create high-quality visualizations of genomic signals. All options are documented with detail in the package documentation and examples are provided.

### Signal plotting regions

recoup creates sequencing signal coverage plots over and around the following genomic region types. In all cases, the profiles can be flanked by a fixed number of bases.

#### Annotated single-point genomic loci

These sites include Transcription Start Sites (TSSs) and Transcription End Sites (TESs). The coverage profiles (average coverage, heatmap, and correlation plots) are centered on the TSS of the genes over which the calculations are made (supported genomes or user-provided genes or gene subsets).

#### Annotated genomic loci of variable length

These sites include gene bodies and 3′ UTRs. The coverage profiles consist of two flanking parts (upstream of the TSS and downstream of the TES for gene bodies or downstream the end of the 3’UTR for 3′ UTRs) and a middle part consisting of the gene body or 3′ UTR coverage profile. The latter are constructed by creating a fixed number of intervals (bins) along each gene/region and averaging the coverage of each interval. These plots are calculated over gene and 3’UTR regions (supported genomes or user-provided genes or subsets).

#### Custom genomic loci

The profile of plotting depends on the custom regions’ length. If it contains single-base intervals (e.g. ChIP-Seq peak centers, Single Nucleotide Polymorphisms), then the profile is similar to that of TSSs. If it contains genomic intervals of equal or unequal size, the profile is similar to that of gene bodies.

### Visualization methodology

The signal calculation methodology, as well as the resulting figure genomic resolution depends on the type of plot to be created. In the case of TSSs, TESs and some cases of custom regions (e.g. SNPs), the signal is calculated based on the read pileup over each base or each bin of bases, depending on the resolution preferred. Then a continuous plot is created based on the signal profile matrix calculated by recoup for each base/bin (columns in the matrix) and each genomic region (rows in the matrix).

In the case of gene bodies or other non-equal custom regions, the signal calculation is split in two phases. Firstly, the signal over the flanking regions (if any) is calculated in the same manner as in TSS/TES. Secondly, the regions of unequal length are split into an equal number of bins for the subsequent creation of the profile matrix. In the case of genomic regions of length larger than the bin size, the procedure of splitting is straightforward. In the case of region lengths smaller than the number of bins, an interpolation procedure must be used to reach the desired bin size and recoup deploys the following strategies:

i) Using smoothing splines. With this option, a spline interpolation of the same size as the number of bins is estimated and is used as the coverage for that region.
ii) Using linear interpolation, as in (i).
iii) Using a nearest-neighbor imputation approach. When selected, a number of missing values are distributed randomly across the small area coverage vector, excluding the first and the last two positions, in order to reach the desired number of bins. Then, each missing value position is filled with the mean value of the two coverage values before and the two coverage values after that position. This method should be avoided when > 20% of the values of the extended vector are missing. However, it should be the most accurate where a few values are missing to reach the desired bin size.
iv) Using a hybrid approach (the default). When chosen, a hybrid approach using splines and nearest-neighbor methods is applied. If the missing values constitute more than 20% of the extended vector, splines are used, otherwise the nearest-neighbor method is used.

## Results and discussion

### Package design and rationale

Driven by the need to quickly create flexible, high-quality, multi-view and potentially publication-ready plots of NGS signals from various protocols such as ChIP-Seq, ATAC-Seq, RNA-Seq and others where binding or expression signal visualization is important, the recoup package was developed. The chosen development framework was the R language for a series of reasons. First of all, Bioconductor comprises an established environment for the fundamental import and processing of NGS data from various protocols with constantly improving speeds, algorithms and architectural concepts like memory footprints. Furthermore, R offers mature mechanisms for the creation of various plot types through the provision of general-purpose packages such as ggplot2 and ComplexHeatmap. These packages along with recoup internal configurations are able to generate high-quality plots for everyday work as well as publication quality graphics. In addition, although Bioconductor offers a wealthy list of various analysis and visualization tools, the comprehensive, flexible and systematic visualization of NGS signals was left behind as existing solutions (CoverageView, SeqPlots) lack flexibility (e.g. accepted file formats, provision of precalculated reference regions, support for various genomic loci types) and user-friendliness.

Most other solutions are developed in languages often disconnected from a more generic analytics framework. For example, seqMINER is written in Java, ngs.plot is a mix of Linux Shell, R and Python, CoverageView and SeqPlots are written in R but are either limited in functionality (CoverageView) or disconnected from the R environment and other packages for analytics (SeqPlots). fluff is written in Python, which apart from a full capable programming language is also a well-established data analytics framework. However, it functions in a way that disconnects it from these capabilities.

Last but not least, there is currently no solution which can create faceted signal plots (average coverage or heatmaps) based on a design or factor provided by the user and potentially based on prior knowledge, without additional labor and multiple runs of a package routines. fluff partially offers this functionality and what is common among these visualization packages is only the provision of hierarchical or k-means clustering of the profiles.

recoup was designed with all the aforementioned points in mind, so as to provide a visualization mechanism which achieves ease-of-use, high-quality plots and connection with other analytics tools (through R and depending on user experience and questions) in an established unified environment at the same time. A typical example of downstream NGS signal analysis which requires connection with a language for analytics is the statistical analysis of signal profiles to discover and verify differences between signals or subsets among them. Within recoup this can be achieved easily by using the package facilities to retrieve the calculated signal matrix and conduct statistical testing within R. In addition, recoup not only achieves faceting elegantly through a very simple text file but also allows for changes on-the-fly within R and operating on the object returned by the main routine.

### recoup plot types

**Fig. 2:**
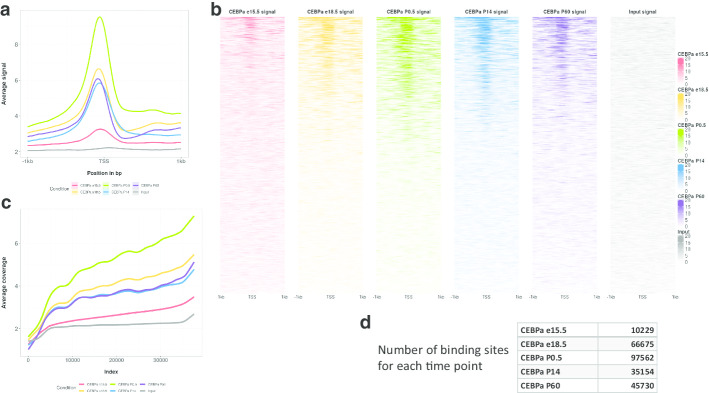
Examples of the different NGS signal plot types provided by recoup applied on the CEBPα dataset from [9]: **a** Average coverage profile of genome-wide CEBPα binding around (1 kb upstream and 1 kb downstream) the Transcriptional Start Sites (TSSs) of all mouse genes as well as the signal of Input DNA used as control, **b** Signal heatmaps depicting the coverage profile of genome-wide CEBPα binding around the TSSs for each gene, ordered from top to bottom according to signal strength, **c** Averaged coverage profiles across 2 kb around the TSS for each gene ordered in ascending order using stage e15.5 as a reference depicting correlation in terms of binding patterns and binding strength across the five time points and the Input, **d** The number of putative binding sites for each mouse liver developmental stage as described in [9]

The recoup NGS signal plotting mechanisms can be used to create three types of signal visualization, each answering to similar questions but from different points of view. Figure 2a-c present the three visualization types applied in ChIP-Seq data from [9] and specifically, the genome-wide monitoring of the transcription factor CEBP/α binding across five developmental stages in mice liver (embryonic days 15.5 and 18.5, Post-natal days 0.5, 14 and 60). A table with the putative binding sites at each time point is also provided for plot interpretation purposes (Fig. 2d). Figure 2a and c are generated with publication quality in mind and require a few more lines of code and recoup option settings. The default images are presented in Supplementary Figure 1A and 1B (Additional File 1). The code used to generate all the figures (main and supplementary) can be found in Additional File 2.

Figure 2a presents the average signal or average coverage profile of genome-wide CEBP/α binding around the Transcriptional Start Sites (TSSs) of all mouse genes as well as the signal from Input DNA used as control. The plot is created by summarizing, normalizing and averaging the read coverage across 1 kb upstream and downstream from the TSSs for each time point and the Input in order to create a genome-wide binding profile of CEBP/α. Although it can be seen that the average signal strength as this is reflected on the vertical axis is proportional to the number of binding sites (Fig. 1d), the plot reveals also that the binding strength as measured by read coverage around the TSS is also increased. If the latter did not happen but there was a decrease instead, the average signals e.g. for stages e15.5, e18.5 and P0.5 would have been at similar levels) as this is the case for stages P14 and P60. On the contrary, the Input coverage around the TSS is nearly flat as expected and serves also as a quality control. An alternative representation of Fig. 1a, where individual profiles are plotted in different panels and therefore signal comparisons are potentially clearer, is presented in Supplementary Figure 1C (Additional File 1).

Figure 2b presents again the coverage profile of genome-wide CEBP/α binding around the TSSs but in this representation the signals are not averaged. Instead, the coverage around the TSS of each gene is depicted as a row in a heatmap, with stronger colors representing more abundant read coverage while faint or white coloring represent little to no read coverage. The heatmaps are ordered from top to bottom based on read coverage abundance. While the average coverage representation in Fig. 2a shows the general profile of binding around the TSS, Fig. 2b shows a more detailed profile, indicating also additional features like an indicative distribution of binding site lengths and the number of bound genes. For example, it can be seen that in the e15.5 stage the number of CEBP/α-bound genes is significantly smaller than P0.5. It should be noted at this point that the aforementioned information regarding bound genes cannot be directly extracted and visualized from Fig. 2d. For instance, even though the number of binding sites is increased in e18.5, this increase could only reflect the addition of binding events on the same (or the same number of) genes as in e15.5. In that case, the heatmap in the second column of Fig. 2b would be similar to the one in the first column regarding the pattern, but with stronger coloring across the first rows. Again, the Input DNA sample shows little to no coverage.

Figure 2c presents the average coverage profile of genome-wide CEBP/α binding around the TSS, but with respect to Fig. 2a, the coverage is not averaged per base-pair for all genes, but across all the 2 kb region for each gene. The resulting values are then sorted in ascending or descending order, based on a reference sample (stage e15.5 in Fig. 2c) and a smoothed curve is created. This representation is particularly useful when the investigator interrogates the ChIP-Seq signals not only in terms of abundance or shape around the TSS but also for correlation between different experimental conditions. In this particular case, it can be seen that the binding signal correlates well between the five developmental stages and the abundance corresponds to the number of binding events at each time point. Although expected in this case, the correlation between experimental conditions is not trivial, as the same kind of plot can be used to interrogate different and possibly antagonizing DNA binding proteins. Finally, the Input sample is also depicted as a quality control.

### recoup usage scenarios

Typical questions asked from NGS datasets resulting from popular protocols are both quantitative and qualitative in nature. For instance, in a ChIP-Seq experiment the quantitative component may translate to questions such as “How many enriched binding regions do I have?”, “What are the binding sites common between TFs A, B, and C” or “How many new binding events do I get after the introduction of a new experimental condition?”. These questions are usually answered by peak calling software and tool suites like bedtools [10]. The qualitative component would include questions such as “What is the average binding profile of TFs A, B, and C over the promoters of all genes?”, “How can I visualize RNA polymerase II pausing in the promoter?” or “What is the average H4K20me1 profile over my expressed and not expressed genes? Does it correlate with RNA activity?”. The latter questions are usually answered by signal visualization tools, often requiring heavy computation and manual curation of the input.

The recoup package was designed to answer qualitative questions and implemented around two main conceptual components. The first is previous experience with a variety of related signal visualization tools. Although they accomplish the goal for which they were designed, this often comes at the cost of time spent in repetitive manual input preparation, managing the output, and repeating similar analyses. When repetitiveness is not predicted in the design of a genomic signal visualization tool, the time and computational overhead may be significant. Moreover, if the interrogated dataset involves many experimental conditions or samples, the data management and process tracking factor is added to the aforementioned setbacks as the users need to carefully keep track of the data transformation, manipulation and preprocessing steps carried out possibly outside a common computational environment where reproducibility can be achieved with less effort. The second component around which recoup was built is to quickly answer qualitative questions simultaneously, while at the same time allowing the researcher to obtain versatile views over the data with minimal labor and easy management, including analysis repeatability.

Therefore, in order to answer qualitative questions such as the ones outlined above, recoup can be used as follows:

- *Q1: What is the average binding profile of TFs A, B, and C over the promoters of all genes?*

The user constructs either a simple list with input BAM files for TFs A, B, C or a text-tab delimited configuration file. This is provided as input to the main recoup routine along with one of the supported genomes (e.g. “mm10”) or a GTF file in the case of non-directly supported organism. The user also tells recoup to create ChIP-Seq profiles over the TSS of the selected organism genes. recoup then will create A, B and C coverage profiles over the TSSs at a base-pair resolution, coverage heatmaps and the correlation profile between A, B and C using A as reference.

- *Q2: How can I visualize RNA polymerase II pausing in the promoter?*

The user deploys either a 3rd party algorithm for detecting RNA PolII pausing in the promoter or uses R/Bioconductor facilities along with a simple metric such as the ratio of the number reads 1 kb upstream and 0.5 kb downstream the TSS to the number of reads 0.5 kb to 2 kb downstream the TSS to locate genes where the normal RNA PolII transcribing activity is paused due to the presence of e.g. a factor inhibiting the activity of the transcription complex. Then, the paused and non-paused gene names are placed in the first column of a text tab-delimited file (or an R data.frame) and the indicators “Paused”, “Non-paused” in the second column. This would then comprise the design file to be provided to the main recoup function, along with a list with the RNA PolII BAM files. The user should choose to plot ChIP-Seq profiles over the gene bodies of the preferred organism. recoup will then construct the requested profiles. An alternative to the above approach is that the user instructs recoup to try and separate paused versus non-paused profiles by performing k-means clustering with k = 2.

- *Q3a: What is the average H4K20me1 profile over my expressed and not expressed genes?*
- *Q3b: Does it correlate with RNA activity?*

To answer Q3a, the user deploys (within or outside R) a tool or a criterion to separate expressed versus non-expressed genes. Then, a design file is constructed similarly to Q2 above and provided to recoup along with the H4K20me1 BAM files and the request to plot ChIP-Seq profiles over gene bodies.

To answer Q3b, the user ranks the RNA activity according to gene expression e.g. from a parallel RNA-Seq experiment. The ranking is then provided to recoup (using the option provided for this purpose) and the latter is instructed to create a coverage correlation plot.

### recoup and related software packages

NGS data analysis tools, whether fundamental such as alignment of the sequenced reads to a reference genome or downstream, such as detection of protein-DNA interactions or inference of differentially expressed genes, apart from the related accuracy in their purpose, they are ranked by execution times and memory footprints. Signal visualization tools are no different in this sense and since the creation of coverage profiles may sometimes be considered trivial, their performance is often measured by computational time or the memory requirements to reach a result. However, nowadays many implementation languages supporting analytics (R, Python) have optimized the underlying algorithms and data structures supporting the analysis of NGS data and therefore minimizing times and memory requirements. Consequently, as execution times tend to become similar among different packages, other factors which contribute indirectly to the time required for the visualization outcomes must be considered. Such factors include complexity of usage, quality of visualization, and data management, in the sense of tracking all the intermediate steps to reach a meaningful visualization under a common computational environment.

Therefore, to compare recoup with existing solutions, the following four combinatorial criteria were defined:

- Visualization, which assessed the quality, flexibility, and information displayed on the output graphs as well as the labor required for manipulation (e.g. adding annotations, changing fonts).
- Reusability, which dealt with issues including the repetition of computationally intensive and time-consuming operations to produce the same plots or a slight variations of similar plots or different views of the same underlying data.
- Flexibility and Friendliness (Flexibility), which scored features such as the inclusion of a GUI and the difficulty levels of using the command line as well as the number of steps to achieve final plots.
- Speed, which measured how fast the results were produced, not only in pure computational time but also in terms of time required to accomplish preparation steps for plotting and managing the output.

In order to qualitatively compare recoup with other packages and pipelines used for NGS signal visualizations (described in Additional File 1, section “Qualitative comparison of recoup with other solutions”), a simple user questionnaire was designed, including a series of questions targeting the evaluation of the aforementioned criteria. The complete questionnaire as well as the answers provided from each user are summarized in Additional File 3. As the number of users was limited (4), they were asked to focus more on their overall experience with each package and therefore, they were not provided with a specific dataset to analyze with each package. Instead, they were asked to apply each package on subsets of their own datasets and also to report what types of NGS data they visualized.

Regarding the Visualization criterion, users were asked to evaluate their experience on a scale between 1 and 10 and intermediate hints were provided to aid their assessment. The intermediate hints were expected outcomes that indicate 1, 5 and 10 in the 1–10 scale. In addition, it was assessed based on on/off criteria according to which visualization types they were able to create with each package. The visualization user experience in general was assessed based on the quality of the output signal visualizations, the formats as well as the number of steps required to achieve the desired views. With respect to the Reusability criterion, users were asked to evaluate in a scale between 1 and 10 the number of steps required to repeat the same analysis as well as create slight variations, such as subset the number of reference genomic loci over which the signals are summarized, always based on their biological questions. As with visualization, hints were provided on the meaning of the rating scale. Furthermore and respecting Flexibility, users were asked a series of questions (on/off as well as rating between 1 and 10) in order to evaluate issues including each package installation process, platform dependence (i.e. multi-platform or only Linux-based), available documentation and tutorials, support for their data and effort spent on preparing the input for each package. The presence or absence of a GUI was also assessed. Finally, concerning Speed, users were asked to rate using a scale between 1 and 10 the time required not only to execute the actual calculations required for each visualization type, but also the time required to prepare the input to each package (e.g. conversion from a file format to another with 3rd party tools, read normalization between samples) including writing configuration files if required.

**Fig. 3:**
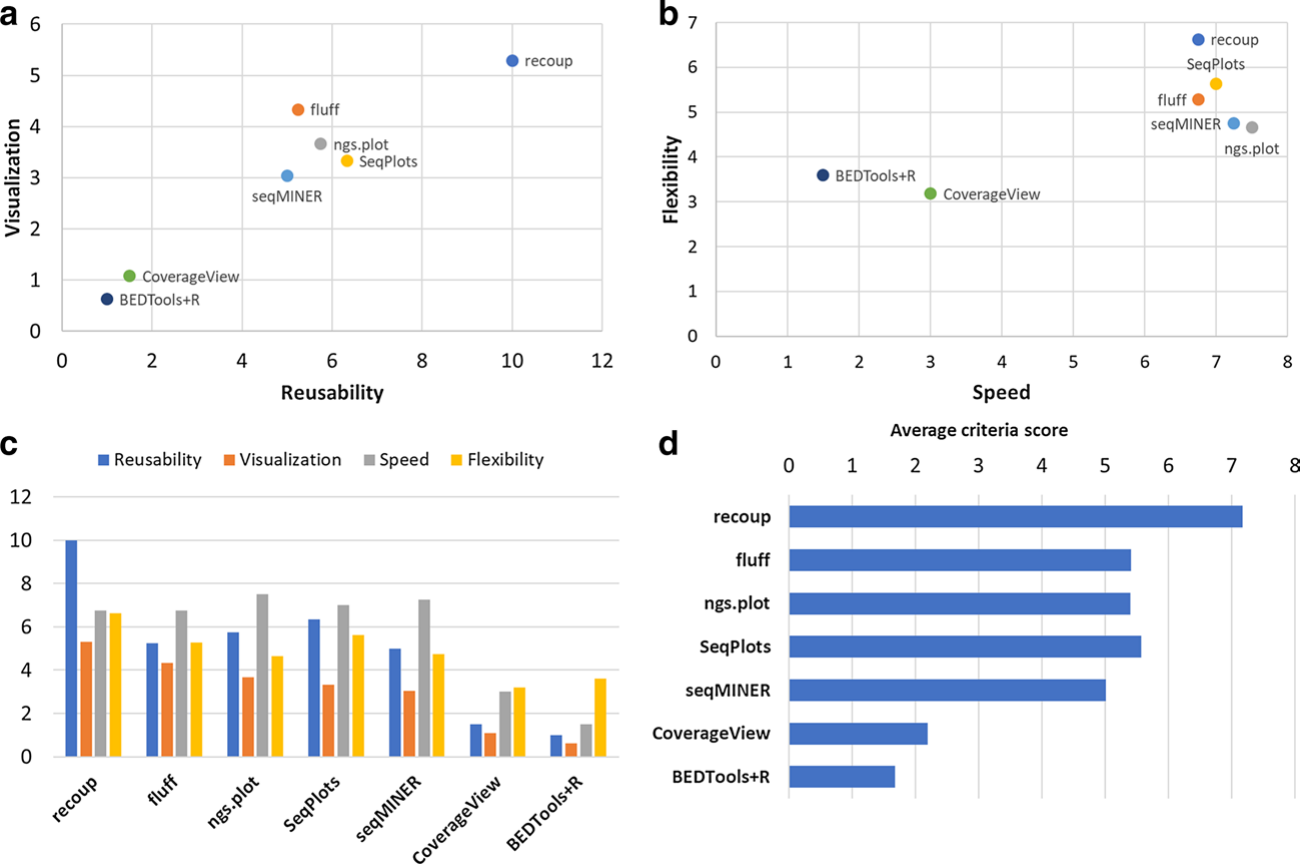
Qualitative assessment of recoup as compared to other popular solutions based on a small user study. **a** Visualization vs Reusability: features including visualization quality and steps to reuse and reproduce the results are assessed. recoup seems to score best in both metrics. **b** Flexibility vs Speed: characteristics such as calculation speed, ease of use, and straightforwardness of procedures are assessed. While recoup is not the fastest tool, mainly due to execution times, it is adequately fast and at the same time offers the greatest flexibility regarding issues like documentation, usability and data preparation. **c** The scores of all four criteria are presented simultaneously instead of pairwise comparisons. recoup excels in most criteria assessed at the same time. **d** The scores of all four criteria presented in the main text were averaged for each package to form a unique combined score. Overall, recoup presents the largest combined score

The scores for each individual question were averaged over the users to form a final score for each question and for each package. Subsequently, the criteria-specific question scores for each package were further averaged to form a unique score for each criterion. For example, Questions 1–4 referred to Flexibility and the final Flexibility score for each package was derived from four values, one for each question. Scores concerning criteria assessed only by one question (e.g. Speed, Question 5) were not averaged. A final combined unique score for each package was constructed again by simply averaging the four individual criteria scores. The limited user study results are presented in Fig. 3. As an additional and complementary way of comparing the various package, a feature matrix was also compiled (Supplementary Table 1, Additional File 1).

Based on user assessment using the aforementioned criteria, the tested solutions were placed in two 2-dimensional coordinate systems (Fig. 3). Figure 3a presents Visualization against Reusability. While most tools achieve the desired answers to biological questions asked, the quality of the visualization as well as fine tuning capabilities and the effort required to achieve these may differ dramatically. Although tools like seqMINER, ngs.plot, fluff, and SeqPlots achieve much of the quality standards of a publication-ready visualization, the way this is achieved may require several steps which cannot be recorded under a unified computational environment. A typical example of this is a series of commands in a Linux shell coupled with a set of configuration files, a setup difficult to maintain and reproduce. recoup appears to be the winner in this qualitative comparison, as it successfully combines visualization capabilities with easy reusability and reproducibility. Figure 3b depicts Flexibility against Speed. While recoup may not be the computationally fastest solution with the lowest memory footprint, as is the case, e.g., with ngs.plot, its inherent flexibility due to reasons mentioned in the Implementation section as well as in the supplementary methods and results (Additional File 1) render it the recommended solution for NGS signal visualization in the longer term.

### recoup use cases

**Fig. 4:**
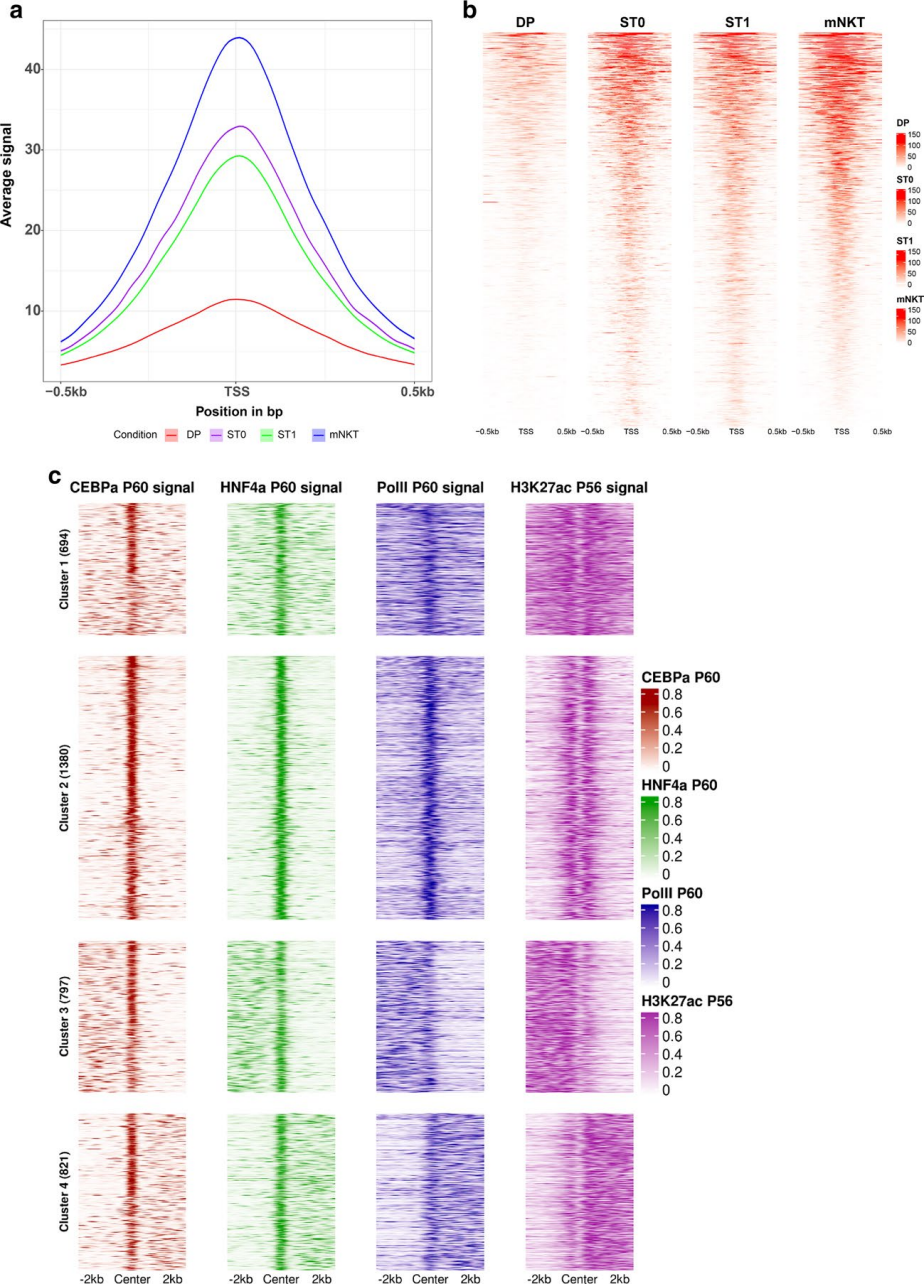
**a** recoup peak center profiles overlaying the DP (red), ST0 (purple), ST1 (green) and mNKT (blue) average chromatin accessibility profiles in iNKT lineage-specific loci. The gradually reducing average read coverage across the TSS indicates the higher accessible chromatin levels from the early stages of invariant NKT commitment. Average signal on the y-axis denotes the average normalized read coverage around the TSS of mouse genes. **b** recoup heatmaps depicting the average chromatin accessibility profile of naive DP thymocytes and the differentiation stages of the iNKT lineage. As in **a**, the gradual reduction of chromatin accessibility is depicted by both the number of genes with accessible chromatin around their TSS as well as the signal strength (red colorbars on the right). With respect to **a**, the heatmaps show detailed normalized read coverage for each gene instead of averaging. **c** Signal heatmap profiles of the transcription factors CEBP/α and HNF4/α, coupled with RNA PolII binding and H3K27ac histone mark signals around the peak centers of the TFs, located in promoters in adult mouse livers. k-means clustering of the TF and H3K27ac profiles reveals regions of potential enhancers as well as different groups of binding strength with respect to transcription and TF co-binding

Apart from routine quality control inspection (Supplementary Figure 2, Additional File 1) and quick interrogation of related data, recoup has been successfully used in several high-impact publications, including the analysis of heterogeneous data under multiple and complex experimental settings requiring advanced signal visualizations. A few examples include:

- Visualization of average accessibility profiles of regions which are more accessible in mature NKT (mNKT) cells compared to Double Positive thymocytes (DP), the state before lineage commitment, using ATAC-seq data of 4 differentiation stages, namely DP, ST0 (Stage 0 T-Cells), ST1 (Stage 1 T-Cells), and mNKT. Results showed that these regions were already more accessible from the early stages of invariant NKT (iNKT) commitment, indicating that chromatin accessibility in lineage-specific loci is mainly determined from early stages of the NKT maturation program [11] (Fig. 4a, b). This result is extracted by the fact that the blue curve corresponding to mNKT cells is both higher and wider, indicating larger, wider and stronger (in terms of read coverage) chromatin accessible sites (Fig. 1a). Accessibility is gradually reduced towards reaching the DP state. The chromatin accessibility signal structure is displayed in more detail in Fig. 4b (heatmap), which depicts genes with ranked ATAC-Seq signal around their TSS. The gradual accessibility decrease can be distinguished by both the number of genes where chromatin is accessible as well as the signal strength (colorbars on the right).
- Visualization of signal heatmap profiles of the transcription factors CEBP/α and HNF4/α, coupled with RNA PolII binding and H3K27ac histone mark signals in promoters in adult mouse livers [9] (Fig. 4c). Such heatmaps, especially when combined with a clustering method such as k-means clustering, are very useful in revealing possible correlations between signals of single as well as various types. In this particular case, the application of k-means clustering on the signal matrices of four ChIP-Seq binding signals (CEBP/α, HNF/α, RNA PolII and H3K27ac histone marks) in adult mice revealed four distinct categories of enhancer activity, as measured by the presence of H3K27ac histone marks. Specifically, cluster 1 shows strong enhancer activity at specific CEBP/α and HNF/α binding sites coupled with increased transcription as measured by RNA PolII levels). Cluster 2 shows less strong activity for H3K27ac and RNA PolII but increased binding strength for CEBP/α and HNF/α. Clusters 3 and 4 show weaker enhancer histone marks around CEBP/α and HNF/α peak centers along with also weaker transcription levels). The exact data analysis procedures and signal interpretations in terms of the underlying genes are described elsewhere [9].
- Visualization of H4K20me1 ChIP-Seq data from post-natal day 45 (P45) wild-type and Kmt5a methylase knock-out mouse livers (Supplementary Figure 2A, Additional File 1) and the overlay of Kdm7b and H4K20Me1 average binding profiles in P45 wild-type mouse livers (Supplementary Figure 2B, Additional File 1) [12].

## Conclusions

The constant expansion of the NGS application space continues to challenge the related analytics and visualization tools. The first contact point of investigators with their NGS data as well as the means to answer several qualitative questions is usually the visualization of the data in genome browsers or other signal visualization tools. Here, we presented recoup, a Bioconductor package for the quick, flexible, versatile, and reusable visualization of NGS signals under complex experimental settings. While existing solutions may offer results relatively quicker and possibly with slightly less computational resources in comparison with recoup, they may incommode users in issues such as tracking of data and processing steps under a common computational environment, visualization quality, and user friendliness, all of them being important features in modern analysis of NGS data. At the same time, while some tools satisfy calculation speed, intuitiveness and include a GUI, they require additional labor before and after the actual visualization process. recoup comprises a package that offers a balanced tradeoff regarding the criteria examined in this article, producing high-quality, flexible, reusable, and easy to manipulate genomic profiles.

## Availability and requirements

**Project name:** recoup

**Project home page:** recoup is an R/Bioconductor package and can be downloaded from https://bioconductor.org/packages/recoup. More examples and documentation can be found in the respective GitHub repository https://github.com/pmoulos/recoup

**Operating system(s):** Linux, Windows, MacOS (platforms where R operates)

**Programming language:** R

**Other requirements:** R version 4.0.0 or higher

**License:** Artistic License 2.0

**Any restrictions to use by non-academics:** The same restrictions as the ones applied to R/Bioconductor usage.

## Supplementary Information

**Additional file 1**. Supplementary material.

**Additional file 2**. Library(recoup): Code used to generate all the figures.

**Additional file 3**. The complete questionnaire and the answers.

## Data Availability

The dataset examined in Fig. 4a and b is reported in [11] with GEO accession number GSE134210. The datasets examined in Fig. 4c are available from GEO with accessions GSE137066 and GSE52386. The dataset examined in Supplementary Figure 3, Additional File 1 is available from GEO with accession GSE97338.

## Abbreviations

NGS: Next Generation Sequencing
GUI: Graphical User Interface
NKT: Natural Killer T-cells
DP: Double Positive thymocytes
ST0: Stage 0 T-cells
ST1: Stage 1 T-cells
TSS: Transcription Start Site
CEBP/α: CCAAT/enhancer-binding protein alpha
HNF4/α: Hepatocyte Nuclear Factor 4 Alpha
