# Supplementary material for: recoup: flexible and versatile signal visualization from next generation sequencing
Source: BMC Bioinformatics. 2021 Jan 6;22:2. doi: 10.1186/s12859-020-03902-x (PMC7789646; doi:10.1186/s12859-020-03902-x)
Supplement: Supplementary file 1 — Additional file 1. Supplementary material. [file 12859_2020_3902_MOESM1_ESM.docx]

**recoup: versatile signal visualization from next generation sequencing**

**Panagiotis Moulos**

Institute for Fundamental Biomedical Research, Biomedical Sciences Research Center ‘Alexander Fleming’, Fleming 34, 16672, Vari, Greece.

Supplementary material

Contents

[Supplementary methods 2](#_Toc56670591)

[Data input and preprocessing 2](#_Toc56670592)

[Input 2](#_Toc56670593)

[Preprocessing 2](#_Toc56670594)

[recoup object and reusability 2](#_Toc56670595)

[Local annotation store 3](#_Toc56670596)

[Basic requirements 3](#_Toc56670597)

[Supplementary results 4](#_Toc56670598)

[Qualitative comparison of recoup with other solutions 4](#_Toc56670599)

[ngs.plot 4](#_Toc56670600)

[seqMINER 4](#_Toc56670601)

[SeqPlots 5](#_Toc56670602)

[fluff 5](#_Toc56670603)

[CoverageView 6](#_Toc56670604)

[BEDTools + R 6](#_Toc56670605)

[Supplementary figures 7](#_Toc56670606)

[Supplementary Tables 9](#_Toc56670607)

[References 11](#_Toc56670608)

# Supplementary methods

## Data input and preprocessing

### Input

The main input to recoup is a list of files with short read information derived from a next generation sequencing protocol. Supported formats are BAM^[[1]](#footnote-1)^, BED^[[2]](#footnote-2)^ and BigWig^[[3]](#footnote-3)^. Obviously, starting from BigWig files would result in the fastest profile generation. However, reads per million plots are not available with BigWig files. The secondary input is either a keyword referring to one of the supported genomes or a user-specified BED-like file with the genomic regions of interest. Optionally, a third input may be provided which comprises a design file to be used for faceting and/or clustering the signal profiles. The input short reads may be provided either as an R list with certain attributes describing the input (name, filename, color etc.) or as a text tab-delimited file where columns represent these attributes.

### Preprocessing

The provided genomic regions are used to mask the input short reads to speed up calculations. A number of flanking bases can be added to each side (5’, 3’) of the provided genomics regions to show potential signal transitions. Prior to masking, the input genomic regions are extended on each side twice the library fragment length or, if the latter is not provided, twice the median read length. The extension is performed in order to avoid signal bleed in the edges of the regions.

After reading, additional optional preprocessing steps follow. The first step is short read cleaning at various levels (e.g. duplicate removal and exclusion of non-anchored to a chromosome sequences to improve speed). The second step is signal normalization which can be performed with the downsampling of the number of reads to the library of the smallest size, downsampling to a specific number of reads or linear normalization (after coverage calculations).

## recoup object and reusability

After each recoup run, an object is returned holding the data generated by the run. This object is a simple R list which contains:

- The data: for each sample, it holds the input short reads ranges (as Bioconductor *GRanges* objects [1]), the calculated coverages over the genomic regions of interest (as Bioconductor *Rle* objects) and the signal profile matrices. The data can be used again as an argument to recoup as well as other package functions.
- The design: the design data frame which is used to facet the profiles.
- The plots: the ggplot2 and/or Heatmap objects created by recoup.
- The recoup call arguments: the majority of recoup call parameters. Their storage serves the reuse of a recoup list object so that only certain elements of plots are recalculated.

Although interoperability and reusability are common among R/Bioconductor packages and functions, the recoup object is particularly designed to serve the aforementioned purposes. This means that only necessary calculations are taking place upon iterative calls that may be executed to fine tune the plots or slightly change some parameters, like for example subsetting the initial set of interrogated genomic regions. The recoup object can be passed among various recoup functions with or without being changed, according to its contents. For example, if the short read input files remain the same, they will not be read again and if the normalization method has not changed between several calls, then the coverages held in the object will also not change, speeding up the total processes. If such recalculations are necessary, then the default behavior of not recalculating what is not necessary according to the recoup mechanisms can be overridden. Finally, to control the output object size, the user can control what is maintained in this object. This is particularly useful in cases where the levels of plot manipulation and analysis experimentation is partly known beforehand.

## Local annotation store

In order to create the signal profiles, a set of genomic regions must be provided, over which coverage is calculated. recoup supports a set of reference genomic regions from widely used genomes and from a variety of sources (Ensembl, RefSeq and UCSC). The recoup supported reference genomic regions are stored in an SQLite database and are versioned for reproducibility reasons. These are:

- Human (*Homo sapiens*) genome versions hg38, hg19, hg18
- Mouse (*Mus musculus*) genome versions mm10, mm9
- Rat (*Rattus norvegicus*) genome versions rn6, rn5
- Fruitfly (*Drosophila melanogaster*) genome versions dm6, dm3
- Zebrafish (*Danio rerio*) genome versions danRer7, danRer10, danRer11
- Chimpanzee (*Pan troglodytes*) genome versions panTro4, panTro5
- Pig (*Sus scrofa*) genome versions susScr3, susScr11
- Horse (*Equus cabalus*) genome version equCab2
- Arabidopsis (*Arabidobsis thaliana*) genome version TAIR10

Apart from the above standard organisms, users can supply own genomic features (or modifications for standard organisms) using a file describing the features in GTF format and respective recoup facilities described in detail in the package documentation. Then, the new annotation is added to the local database and can be used in a recoup call like the standard annotations.

Pre-calculated genomic region coordinates include:

- Gene bodies
- 3’ UTRs
- Exons, with their respective gene starts/ends flanked by 0.5, 1, 2 and 5kbs

If the local region database is not present, all annotations can be retrieved on-the-fly. However, in some cases this is inefficient. The regions database is also available for download^[[4]](#footnote-4)^ and new versions are added periodically.

## Basic requirements

In order to create recoup visualizations, two main inputs are required:

- A set of BAM, BED or BigWig files that contain the aligned short reads from a DNA sequencing experiment (e.g. ChIP-Seq from transcription factors, DNA methylation signals, DNAse-Seq, ATAC-Seq etc.) or RNA sequencing experiments (RNA-Seq, spliced/unspliced). Theoretically, alignments from most protocols can be used with recoup, for example to measure and visualize the average coverage in Whole Exome or Gene Panel sequencing over the capture targets.
- A set of reference genomic regions over which to calculate profiles. These can be provided as a BED-like text file with a header. They can also be provided as an organism version/code (e.g. hg19, mm10) and the respective regions will be either retrieved from the local annotation database setup, or downloaded on the fly (may takes more time as additional operations are required).

Optionally, a design file can be also provided for further categorization and faceting of the profiles. Examples of such categories may be:

- A set of H3K27me1 or H4K20me1 profiles categorized by gene transcription or expression levels – high, medium, low
- Different levels of binding strength of a transcription factor close to the TSS
- Different levels of gene expression to be associated with open accessible chromatin

The design file should have in the first column unique identifiers which should be the same, a subset or a superset of those in the reference genomic regions. These identifiers can be gene names or identifiers of peak regions in a ChIP-Seq experiment for example.

# Supplementary results

## Qualitative comparison of recoup with other solutions

Since their introduction, next generation sequencing protocols continue to generate a great number of datasets which also continue to grow in size. Therefore, the visualizations of the native signal generated by the short reads is essential for the understanding of underlying biological mechanisms, as they comprise intuitive tools for summarizing this category of big biological data. As a result, a number of tools have been proposed in the literature over the past decade. A selection of these tools was made, based mostly on popularity, in order to make a qualitative comparison with recoup and namely, seqMINER [2], CoverageView^[[5]](#footnote-5)^, ngs.plot [3], fluff [4] and SeqPlots [5]. In addition, a simple scenario of using BEDTools [6] to calculate coverage over genomic regions of interest and then R to create simple plots over these regions by reading and manipulating the respective output files from BEDTools, is included as a reference of a basic procedure used to create signal profiles without the inclusion of specific tools designed for this purpose.

### ngs.plot

One of the most comprehensive tools is ngs.plot. It is sufficiently fast for most applications and has a low memory footprint. Also, it runs in command line, most times based on a simple configuration file, has a rich database of genome annotations and uses R/Bioconductor for underlying calculations and plotting of profiles. However, ngs.plot is not up to date with modern R graphics systems like ggplot2. As a result, it is impossible to create faceted genomic profiles using a statistical design, requiring loads of additional manual work and time to reach the desired outcomes. Furthermore, the resolution of genomic profiles (e.g. per base coverage or exact binning of bases) cannot be controlled and this can be problematic in cases where high resolution is required (e.g. DNAse-Seq experiments) to reach meaningful biological conclusions. Last but not least, ngs.plot requires a non-straightforward setup in order to run, does not run in a unified working environment (e.g. the R language environment with embedded graphics manipulation mechanisms) and produces complex output.

With respect to ngs.plot, recoup offers a straightforward mechanism for creating high-quality and high-configurable plots in a unified working environment, while at the same time being quite fast. Furthermore, recoup supports more visualization options, requires substantially less time to prepare the inputs, supports more input formats and eases the repetition of particular analyses. Finally, both tools support a pre-defined database of genomic regions to speed-up analyses for widely used model organisms, although recoup supports more organisms.

### seqMINER

One of the first tools introduced for the visualization of next generation sequencing signals with a specific focus initially to ChIP-Seq experiments and then additions for RNA-Seq has been the seqMINER package. seqMINER offers a GUI and is quite fast in terms of loading data, signal calculations, visualization rendering and color selection. It also offers facilities to perform k-means clustering. However, its usage is not entirely straightforward despite the GUI and the input data may require substantial processing and manipulation as it operates with BED files. Furthermore, it does not offer the option to save intermediate work, rendering the reproducibility of an analysis or its repetition with slight changes often a tedious task. In addition, its visualization mechanisms are rather old and they focus more on the generation of heatmaps and less to average coverage signals. Finally, it does not support operations like ordering of signal and normalization within the software and any similar manipulations have to be performed manually outside the program and it is not shipped with a database of genomic regions.

Instead, recoup offers many more functionalities when compared to seqMINER, including support for a variety of input formats through optimal usage of Bioconductor facilities, signal operations, reusability, plotting flexibility and a rich database of genomic regions ready to use. Finally, recoup does not have a GUI, something which is present in seqMINER.

### SeqPlots

SeqPlots is an advanced tool for visualizing genomic signals in average coverage and heatmap formats and also supports functionalities for visualizing DNA motif densities in the case of e.g. ChIP-Seq experiments. Although it ships with a GUI, can also be used in command line mode and belongs to the Bioconductor repository, it presents certain lacks in flexibility, options for plot rendering and manipulation as well as supported genomic region databases. In addition, although it supports multiple short read input file formats, the preferred format is BigWig. Although BigWig files offer the fastest solution for signal calculation and plot rendering, they do not offer the maximal possible resolution as the signal derived directly from short reads has already gone through a round of summarization over fixed bins of bases. Furthermore, functions like plot zooming, dataset slicing and other related element of reusability are not present.

On the other hand, the preferred file format in recoup is BAM in order to maximize the available signal plot resolution but BED and BigWig formats are also supported. Although recoup does not ship with a GUI, all its major functionalities are offered through a small number of functions, the learning of which presents a rather small overhead when put in context of flexibility, reusability and plot quality. Finally, the installation of recoup is straightforward: firstly, the package is installed through Bioconductor and secondly, the recoup database can be built with executing just one function or downloaded from a pre-built source. On the contrary, SeqPlots has been reported to present certain problem in installation under circumstances^[[6]](#footnote-6)^.

### fluff

fluff is another advanced, command line only, visualization framework for the exploration of next generation sequencing genomic signals. It is written in Python, a fact that already makes it somewhat hard to use for a category of less experienced users as it requires some prior knowledge on Python versioning and packaging system, as well as potentially system invasive frameworks such as bioconda^[[7]](#footnote-7)^. This level of complexity is reinforced by the fact that it has some dependencies outside the common computational framework of Python. In addition, to the best of our knowledge, it does not ship with or has a method to create a predefined database of genomic regions as compared for example with ngs.plot, it has a limited set of plot tuning and parameterization options – which is adequate for most users though – and not a clear functionality for saving intermediate work and reproducible plotting without repeating all the work. Notably, it has a mode for creating genome browser like signal profiles (i.e. not averaged across genomic regions) of publication quality, a functionality difficult obtained through classic genome browsers and rare in similar tools.

In comparison with fluff, recoup is easier to set up and run as a Bioconductor package within a common computational framework (R) and it supports many more visualization and within-package data processing options, but has a larger memory footprint. It also comes with a rich set of predefined genomic regions for the user to map the data over and plots are much more customizable in real-time (i.e. not post-processing through an image processing software). Finally, recoup does not create genome browser-like profile instances. This is included in future work through for example the integration of the ggbio package [7].

### CoverageView

CoverageView is a simple Bioconductor package for reading short read data and creating basic visualizations of genome sequencing signal profiles in coverage and heatmap formats. The package is very basic in its functionalities which means that it creates only basic views not suitable for publication, does not include any predefined genomic regions, does not support many options for visualization outside standard R graphics. Also it does no offer a framework for saving intermediate work or analysis reproducibility, does not work with a variety of input file formats and does not include any GUI. However, it has an interesting feature which is the juxtaposition of average coverage profiles and heatmap profiles for inspection at the same time. Given that there are more advanced packages supporting this (e.g. SeqPlots), although interesting, this feature is not unique and the views continue to be basic.

recoup supports all the aforementioned functionalities and as mentioned during the qualitative comparison analysis of this sections, many more useful and versatile functionalities. However, recoup does not support the double view of coverage profile and heatmap.

### BEDTools + R

For very experienced users that want to create one-time visualizations, both simple and complex, the sequential usage of multiple packages to create the final output may be ideal. There are no bindings to particular platforms other than the operating system and there is also the freedom of using and testing multiple tools and plotting systems to achieve the ideal outcome. Moreover, the raw signal data can be processed with additional statistical algorithms to extract potentially hidden biological insights which cannot be revealed using any of the aforementioned visualization frameworks. Therefore, one could use BEDTools to read, process, interrogate and manipulate raw short read files and create a fine-grained answer to a particular biological question and then read the resulting files into R and create visualizations as well further process data using a variety of packages. However, this ultimate flexibility, apart from bioinformatics training and experience, requires large amounts of time to set up and identify the required packages as well as definitely larger times to maintain.

Nevertheless, it is clear that such solutions may not be preferred or sustainable, as if it was the case, no specialized tools for sequencing signal visualization would have been developed. recoup offers an optimized tradeoff between the ultimate freedom and flexibility that the aforementioned combined solutions offer and the computational and time burden required to setup and maintain them, while at the same time maintaining the flexibility required by the vast majority of users. In addition, it lives within a well-defined, popular and maintained ecosystem (Bioconductor) ensuring consistency, robustness and sustainability.

# Supplementary figures


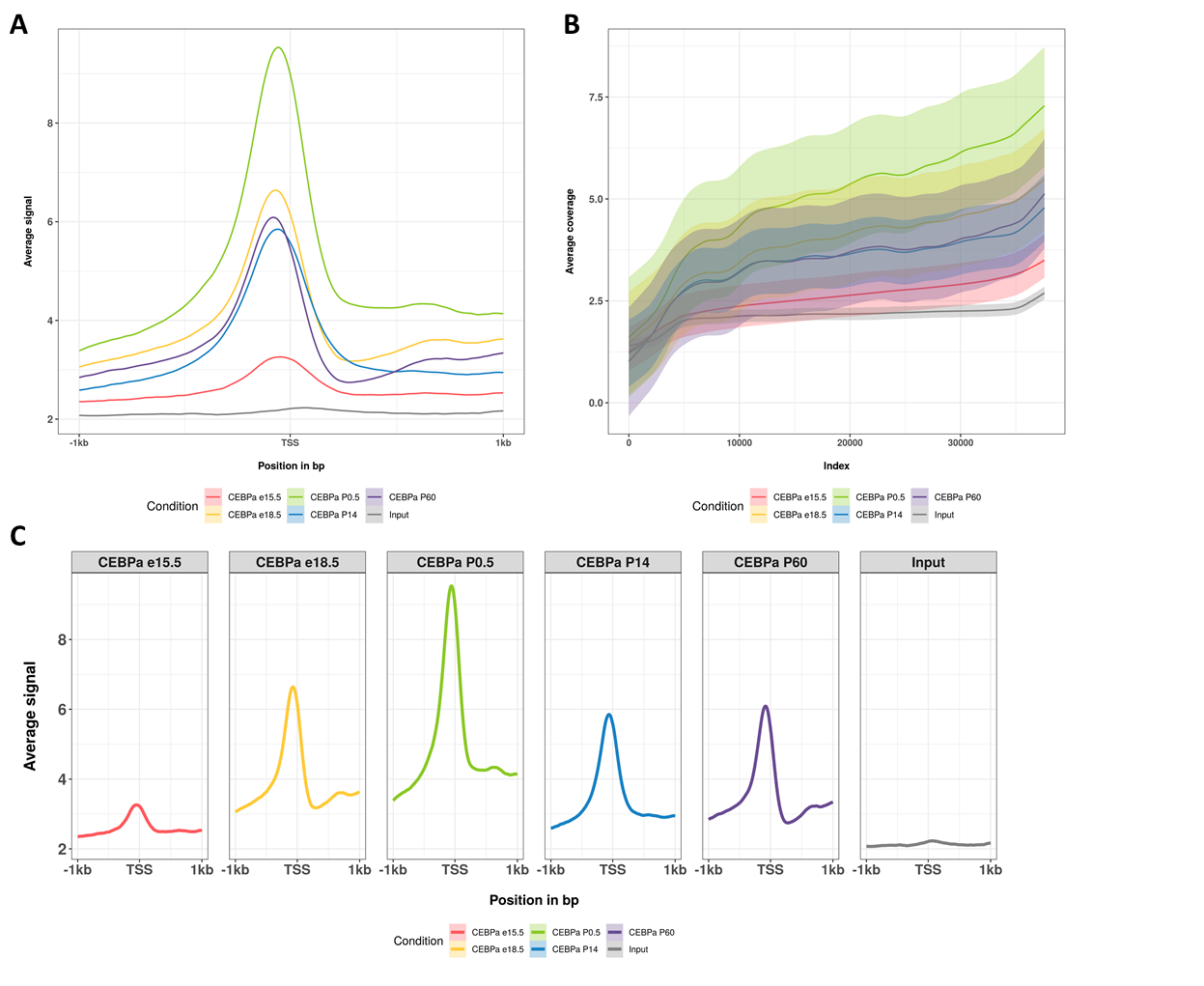


**Supplementary Figure 1**: Examples of recoup average signal and correlation plots as they are directly produced by the package for inspection, without additional options applied to produce publication-ready figures. The example dataset is discussed in the main text.


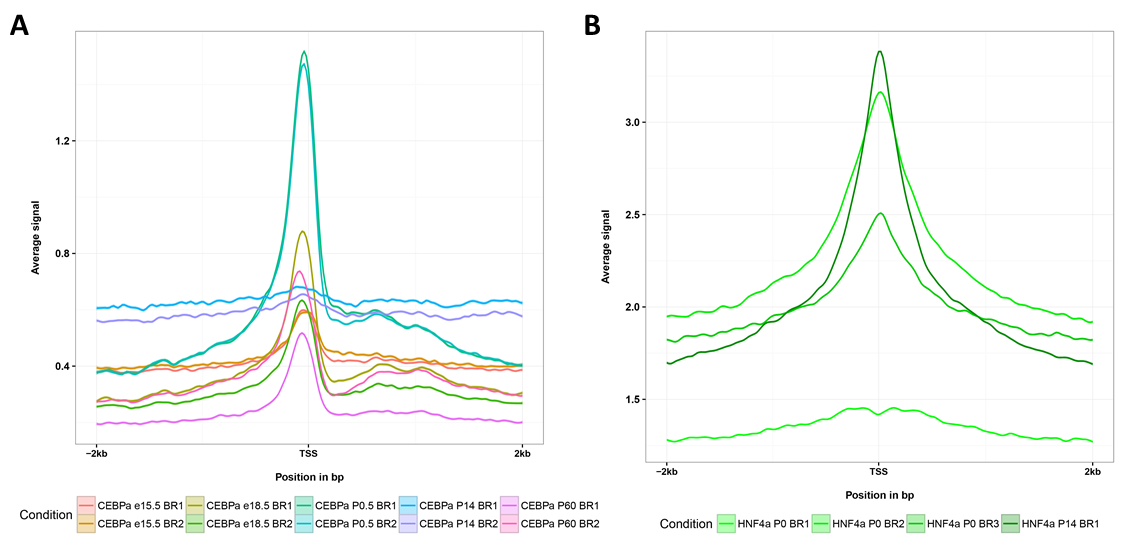


**Supplementary Figure 2**: Examples of quality control checks performed with recoup using sequencing runs to produce the data from [9]. A. CEBP/α transcription factor ChIP-Seq coverage profiles around the TSS of all mouse genes for five mouse liver developmental stages with two replicates each. While for stages e15.5, e18.5, P0 and P60, a significant signal rise can be observer around the TSS, as expected for transcription factor profiles, the signal for P14 is close to flat, indicating experimental failure and that the experiment should be performed again. B. Similar profiles for the transcription factor HNF4/α. The 1^st^ replicate at P0 shows failure.


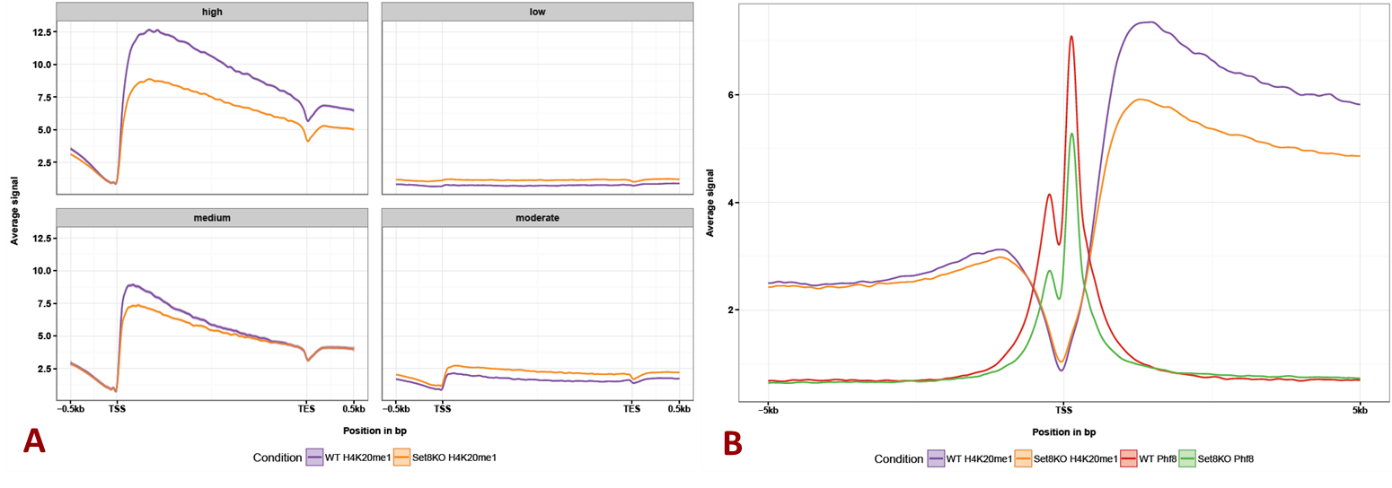


**Supplementary Figure 3**: *A*. recoup gene body average coverage profiles of H4K20me1 ChiP-Seq data from P45 WT and Kmt5a methylase (also known as Set8) KO mouse livers. The H4K20me1 profiles are categorized by the respective gene expression categories (high, medium, moderate, low) indicating high-levels of correlation between gene expression and H4K20me1 binding levels. H4K20me1 levels drop upon KO of Kmt5a indicating its role in H4K20me1 binding and underlying gene activity. *B*. Overlay of Kdm7b (red WT, green Kmt5a KO) with that of H4K20Me1 (purple WT, orange Kmt5a KO) average ChIP-seq profiles in P45 wild-type mouse livers. Kdm7b (also known as Phf8) demethylase is the antagonist of Kmt5a methylase [8].

# Supplementary Tables

|  | **Recoup** | **fluff** | **ngs.plot** | **SeqPlots** | **seqMINER** | **CoverageView** | **BEDTools+R** |
| --- | --- | --- | --- | --- | --- | --- | --- |
| **Input data types** | BAM, BED, BigWig, GTF | BAM, BED, Wig, BigWig, BedGraph | BAM | BED, BedGraph, Wig, BigWig, GFF | BAM, BED | BAM | BAM, BED |
| **Input signal files definition** | R list, targets file | command line list | single file or from configuration file | file list through the GUI | file list through the GUI | R command line | command line |
| **Plot types** | average coverage profiles, coverage heatmaps, coverage correlations | average coverage profiles, coverage heatmaps, single locus profiles | average coverage profiles, coverage heatmaps | average coverage profiles, coverage heatmaps | average coverage profiles, coverage heatmaps | average coverage profiles, coverage heatmaps | average coverage profiles, coverage heatmaps or others by using R directly |
| **Plot regions** | continuous, non-continuous | continuous, non-continuous | continuous, non-continuous | continuous | continuous, non-continuous | continuous | continuous |
| **Plot mode** | coverage, rpm | coverage | coverage | coverage | coverage | coverage | coverage |
| **Coverage resolution** | base pair, variable size bins | variable size bins | fixed size bins | depends on input file (e.g. BigWig) | fixed size bins | fixed size bins | base pair, variable size bins |
| **Supported genomic loci** | TSS, TES, gene body, exon, 3’ UTR, custom | custom | TSS, TES, gene body, exon, cgi, dhs, enhancer, custom | TSS, gene body, custom | custom | custom | custom |
| **Precalculated reference loci** | TSS, TES, gene body, 3’ UTR, exon, custom | none | TSS, TES, gene body, exon, cgi, dhs, enhancer | TSS, custom | none | none | none |
| **Profile clustering** | k-means, hierarchical, external design | k-means, hierarchical, external design | k-means, hierarchical | k-means, hierarchical, SOM | k-means | no | no |
| **Profile faceting** | yes in all plots | yes to some plots | no | no | no | no | no |
| **Heatmap ordering** | done internally in ascending or descending order, by reference sample, by total average or max signal, by sample-specific average or max signal, by external reference | done externally | done internally in descending order, by first sample, by product of samples, by difference between 1^st^ and 2^nd^ sample | done internally in ascending or descending order, by mean signal across samples | done externally | no | no |
| **Normalization** | downsampling to lowest library or to specified read number, linear, none | downsampling, reads per million, none | reads per million | none | none | none | none |
| **GUI** | no | no | no | yes | yes | no | no |
| **Profiles as reusable objects** | yes | no | no | yes | no | no | no |
| **Plots parameterization** | yes, extended through package options or ggplot2 | yes, restricted | yes, restricted | yes, extended | no | no | depending on R usage |
| **Genomic annotation databases** | ensembl, refseq, ucsc for 9 organisms, custom from GTF | no | ensembl, refseq for >20 organisms | custom from GTF | no | no | no |
| **Parallelization** | yes | no | yes | yes | yes | no | no |
| **Other** | read filtering at many levels, easy profile subsetting, zooming, merging and slicing, stranded plots, different binning for main and flanking regions, advanced profile interpolation, imputation of missing values, heatmap color-scales, log_2_ transformation | identification of dynamics between different time points or conditions, read filtering at some levels, bandplots | read filtering at some levels, option for defining flanking region size, heatmap color-scales, input data chunking | extended plot and graphics options through the GUI | reordering of clustered profiles by drag and drop, control of color saturation | heatmaps and profiles in single plot | can be a highly flexible pipeline for experienced users |

**Supplementary Table 1:** Summary of features of each reported package for NGS signal visualization.

#

# References

1. Lawrence M, Huber W, Pages H, Aboyoun P, Carlson M, Gentleman R, Morgan MT, Carey VJ: **Software for computing and annotating genomic ranges**. *PLoS computational biology* 2013, **9**(8):e1003118.

2. Ye T, Krebs AR, Choukrallah MA, Keime C, Plewniak F, Davidson I, Tora L: **seqMINER: an integrated ChIP-seq data interpretation platform**. *Nucleic acids research* 2011, **39**(6):e35.

3. Shen L, Shao N, Liu X, Nestler E: **ngs.plot: Quick mining and visualization of next-generation sequencing data by integrating genomic databases**. *BMC genomics* 2014, **15**:284.

4. Georgiou G, van Heeringen SJ: **fluff: exploratory analysis and visualization of high-throughput sequencing data**. *PeerJ* 2016, **4**:e2209.

5. Stempor P, Ahringer J: **SeqPlots - Interactive software for exploratory data analyses, pattern discovery and visualization in genomics**. *Wellcome open research* 2016, **1**:14.

6. Quinlan AR, Hall IM: **BEDTools: a flexible suite of utilities for comparing genomic features**. *Bioinformatics* 2010, **26**(6):841-842.

7. Yin T, Cook D, Lawrence M: **ggbio: an R package for extending the grammar of graphics for genomic data**. *Genome biology* 2012, **13**(8):R77.

8. Nikolaou KC, Moulos P, Harokopos V, Chalepakis G, Talianidis I: **Kmt5a Controls Hepatic Metabolic Pathways by Facilitating RNA Pol II Release from Promoter-Proximal Regions**. *Cell reports* 2017, **20**(4):909-922.

9. Karagianni P, Moulos P, Schmidt D, Odom DT, Talianidis I: **Bookmarking by Non-pioneer Transcription Factors during Liver Development Establishes Competence for Future Gene Activation**. *Cell reports* 2020, **30**(5):1319-1328 e1316.

1. <https://samtools.github.io/hts-specs/SAMv1.pdf> [↑](#footnote-ref-1)
2. <https://genome.ucsc.edu/FAQ/FAQformat.html#format1> [↑](#footnote-ref-2)
3. <https://genome.ucsc.edu/FAQ/FAQformat.html#format6.1> [↑](#footnote-ref-3)
4. [recoup downloads](https://drive.google.com/drive/folders/1CRl0ScNKz7Ai0Kg_FkrCMjad10MPBV8z?usp=sharing) [↑](#footnote-ref-4)
5. <https://bioconductor.org/packages/release/bioc/html/CoverageView.html> [↑](#footnote-ref-5)
6. <https://wellcomeopenresearch.org/articles/1-14/v1> [↑](#footnote-ref-6)
7. <https://bioconda.github.io/> [↑](#footnote-ref-7)
